# Trevo 3 Mm and/or AXS Catalyst 5 for the Treatment of Medium Distal Vessel Occlusion Stroke—results from the ASSIST Registry

**DOI:** 10.1007/s00062-024-01450-y

**Published:** 2024-08-23

**Authors:** Nikolaos Ntoulias, Alex Brehm, Salvador Miralbés, Bharath Naravetla, Alejandro Spiotta, Christian Loehr, Mario Martínez-Galdámez, Ryan McTaggart, Luc Defreyne, Pedro Vega, Osama O. Zaidat, Lori Lyn Price, David S. Liebeskind, Markus Möhlenbruch, Rishi Gupta, Marios-Nikos Psychogios, ASSIST Investigator

**Affiliations:** 1 Diagnostic and Interventional Neuroradiology, University Hospital Basel, Basel, Switzerland; 2 Neuroradiology, Hospital Son Espases, Mallorca, Spain; 3 Interventional Neurology, McLaren Regional Medical Center, Flint, MI USA; 4 Interventional Neurology, McLaren Regional Medical Center, Macomb, MI USA; 5 Neurosurgery, Medical University of South Carolina, Charleston, SC USA; 6 Radiology and Neuroradiology, Klinikum Vest Recklinghausen, Recklinghausen, Germany; 7 Interventional Neuroradiology/Endovascular Neurosurgery, Hospital Clínico Universitario de Valladolid, Valladolid, Spain; 8 Interventional Neuroradiology, Rhode Island Hospital, Providence, RI USA; 9 Vascular and Interventional Radiology, Ghent University Hospital, Ghent, Belgium; 10 Radiology, Hospital Universitario Central de Asturias-HUCA, Oviedo, Spain; 11 euroscience Department, Bon Secours Mercy Health St. Vincent Medical Center, Toledo, OH USA; 12 Stryker Neurovascular, Fremont, CA USA; 13 Department of Neurology and Comprehensive Stroke Center, David Geffen School of Medicine, University of California, Los Angeles, Los Angeles, CA USA; 14 Neuroradiology, Uniklinik Heidelberg, Heidelberg, Germany; 15 Wellstar Medical Group, Neurosurgery, WellStar Health System, Marietta, GA USA; 16 Department of Neuroradiology, University Hospital Basel, Petersgraben 4, 4031 Basel, Switzerland

**Keywords:** Ischemic stroke, Medium vessel occlusion, Distal vessel occlusion, Endovascular therapy, Mechanical thrombectomy

## Abstract

**Background:**

The effect of endovascular therapy (EVT) on the outcome of stroke patients with a medium distal vessel occlusion (MDVO) is unclear. We report the results of MDVO patients treated with the 3 mm Trevo stent retriever (SR) and/or the AXS Catalyst 5 distal access catheter.

**Methods:**

Data was derived from a prospective, multicenter global registry (ASSIST registry) which enrolled patients treated with operator preferred EVT technique at 71 sites from January 2019 to January 2022. Three techniques were assessed: SR classic, direct aspiration, and a combined approach. Additional inclusion criteria were (a) EVT performed with the 3 mm Trevo SR and/or AXS Catalyst 5 distal access catheter on the first pass and (b) an occlusion of the M2 segment or M3 segment of the middle cerebral artery or the A1, A2 or A3 segment of the anterior cerebral artery. The primary outcome was achieving an expanded Thrombolysis in Cerebral Infarction (eTICI) score of 2c or 3 on the first pass, with the primary technique as adjudicated by core lab. The primary clinical outcome measure was a 90-day modified Rankin Scale (mRS) score of 0–2.

**Results:**

A total of 155 patients (10.4% of the ASSIST population) were included. Most patients had an M2 occlusion (93.5%). First pass eTICI reperfusion was achieved in 43.1% of the patients. No modifying effect of the frontline technique was found. The rate of mRS 0–2 (overall 65.0%) did not significantly differ between groups.

**Conclusion:**

The data suggests that the Trevo 3 mm SR and/or the AXS Catalyst 5 may be an option to treat medium distal vessel occlusion, but more data is needed to demonstrate safety and efficacy in this patient cohort. Further improvements are needed regarding materials and techniques to improve reperfusion results in this patient cohort in the future.

## Introduction

Endovascular therapy (EVT) significantly improves the odds for a good functional outcome in patients with large vessel occlusions of the internal carotid artery (ICA), the M1 segment of the middle cerebral artery (MCA) and the basilar artery (BA) [1, 2]. While there is some evidence derived from a patient level meta-analysis of multiple endovascular stroke trials (HERMES) that EVT might be beneficial in patients with an occlusion of the M2 segment (Odds ratio for modified Rankin Scale [mRS] of 2 in favour of EVT 2.68; 95%-Confidence Interval (CI) 1.04–4.81) [3], there is no evidence for other more distal vessel occlusions (namely the M3 or M4 segment of the MCA, the A1, A2 or A3 segment of the anterior cerebral artery (ACA) or the P1, P2 or P3 segment of the posterior cerebral artery (PCA)). Current European Stroke Organisation (ESO) and American Heart Association/American Stroke Association (AHA/ASA) guidelines do not give clear recommendations for or against EVT in patients with Medium Distal Vessel Occlusion (MDVOs) [4, 5].

There are currently several large scale RCTs, such as DISTAL (NCT05029414), DISTALS (NCT05152524), DISCOUNT (NCT05030142), and ESCAPE-MeVO (NCT05151172), actively recruiting participants to address this crucial clinical question. As of now only retrospective studies have addressed this question, showing in most cases neutral effects of EVT on the long-term clinical outcome or minor positive effects on certain endpoints [6–10].

In clinical practice MDVOs are increasingly identified as a target for EVT despite limited evidence. This highlights the need for optimizing EVT approaches and gathering data on technical efficacy and safety of different EVT techniques. The primary focus of this paper is to present clinical and patient characteristics, as well as efficacy and safety outcomes, in patients who underwent EVT using the 3 mm Trevo NXT or XP stent retriever and/or AXS Catalyst 5 distal access catheter on the first pass for treatment of MDVO in the ASSIST registry.

## Methods

### Study Design and Participants

The data for our analyses were gathered from the ASSIST registry, a multinational and multicenter initiative to collect data on patients with acute ischemic anterior circulation large vessel occlusion strokes. Seventy-one clinical sites across 11 countries took an active part in the registry, enrolling a total of 1492 patients. Before patient recruitment, each site gained written approval from the Ethics Committee/Institutional Review Board. This study is registered on ClinicalTrials.gov (registration number NCT03845491). The authors wrote this manuscript according to the STROBE (Strengthening the Reporting of Observational Studies in Epidemiology) cohort reporting guidelines.

In addition to the requirement for presence of acute ischemic stroke (AIS) with occlusion in an intracranial anterior circulation vessel, interventionalists were required to use Stryker Neurovascular approved products on the first pass. Patients or their authorized representatives provided informed consent before or within 48 h after the procedure.

Three techniques of thrombectomy are of interest: stent retriever (SR) with a balloon guide catheter (BGC), a stent retriever combined approach (CA) and direct aspiration without stent retriever (DA). A dedicated independent core laboratory evaluated baseline imaging to identify the initial occlusion site and evaluated angiograms to assess reperfusion grades after each pass and the final pass. See Gupta et al. 2024 for additional information on study design and outcomes for the ASSIST Registry [11].

In this analysis patients were included if they (a) were included in the ASSIST registry, (b) were treated with the 3 mm Trevo NXT or XP stent retriever in combination technique or AXS Catalyst 5 alone on the first pass and (c) had a core lab reported primary occlusion of the M2 segment or M3 segment of the MCA or the A1, A2 or A3 segment of the ACA.

### Device Specifications

The 3 mm Trevo NXT is an SR with a 3 mm diameter, 32 mm labeled length (stent length of 36 mm) and overall device length of 200 cm with a 0.015″ wire. Platinum markers at the distal end allow fluoroscopic visualization. In addition, the shaped section is also radiopaque. The Retriever has a hydrophilic coating to reduce friction during use. The Retriever has a shaft marker to indicate proximity of Retriever tip relative to Microcatheter tip.

AXS Catalyst is a distal access catheter with inner diameter of 1.47 mm (0.058″), designed with an outer diameter of 1.86 mm (0.073″) on its proximal end, tapering to an outer diameter of 1.76 (0.069″) on the distal end. Its effective length, depending on the version, varies from 115 to 132 cm and on its overall length from 120 to 137 cm. The AXS Catalyst 5 Distal Access Catheter is a single-lumen, variable-stiffness catheter designed for use in facilitating the insertion and guidance of appropriately sized interventional devices into the neurovascular system. The catheter shaft has a hydrophilic coating to reduce friction during use. The catheter includes a radiopaque marker on the distal end for angiographic visualization and a luer hub on the proximal end allowing attachments for flushing and aspiration.

### Primary Endpoint

The primary procedural endpoint was the proportion of patients achieving an expanded thrombolysis in cerebral infarction (eTICI) score of 2c or 3 on the first pass to treat the target occlusion with the primary technique as adjudicated by the independent core lab.

### Secondary Endpoints

Secondary procedural outcomes include the proportion of patients achieving a final eTICI score of 2c or 3, a first pass eTICI score of 2b or better and the number of passes. Other secondary outcomes of interest are favorable outcome at 90 days (mRS 0–2) as well the National Institute of Health Stroke Scale (NIHSS) Score at 24 h. Safety outcomes included the proportion of patients with a symptomatic intracranial hemorrhage (sICH) within 48 h, device and/or procedure related serious adverse events (SAEs) at 90 days, and all-cause mortality at 90 days.

### Statistical Methods

Frequencies and percentages are presented for categorical variables and means and standard deviations are presented for continuous variables. *P*-values were generated using generalized linear mixed models with a random effect for continuous variables and chi-square tests adjusted for clustering for categorical variables to account for clustering within center. In cases where the algorithm would not converge, an unadjusted Fisher Exact test was used. *P* < 0.05 is considered statistically significant. SAS 9.4 (Cary, NC) was used for all analyses.

## Results

From the initial 1492 patients 155 met the inclusion criteria for this analysis. The baseline characteristics are summarized in Table 1. Mean age was 68.9 (SD 14.4) years, and 71 patients (46%) were female. The most common comorbidities were hypertension (73%), dyslipidemia (48%) and atrial fibrillation (31%).

**Table 1:** Baseline data

	Overall (*n* = 155)	SR plus BGC (*n* = 5)	Direct Aspiration (DA) (*n* = 34)	Combined Approach (CA) (*n* = 116)
**Demographics, Medical history, Medications**				
Age (years), mean (SD)	68.9 (14.4)	60.6 (14.9)	68.6 (12.5)	69.4 (14.8)
Female, *n* (%)	71 (45.8)	3 (60.0)	14 (41.2)	54 (46.6)
Hypertension, *n* (%)	109 (73.2)	3 (60.0)	24 (70.6)	82 (74.5)
Congestive heart failure, *n* (%)	11 (7.7)	2 (40.0)	4 (12.1)	5 (4.8)
Coronary artery disease, *n* (%)	35 (24.3)	0 (0.0)	6 (18.2)	29 (27.4)
Atrial fibrillation, *n* (%)	46 (30.7)	1 (20.0)	8 (23.5)	37 (33.3)
Previous history of known ICAD, *n* (%)	9 (6.4)	0 (0.0)	5 (14.7)	4 (3.9)
Extracranial carotid artery disease, *n* (%)	11 (7.6)	0 (0.0)	2 (5.9)	9 (8.6)
Diabetes mellitus, *n* (%)	30 (20.7)	2 (40.0)	9 (26.5)	19 (17.9)
Dyslipidemia, *n* (%)	67 (47.5)	3 (60.0)	17 (50.0)	47 (46.1)
*Smoking Status, n (%)*				
Never	75 (60.0)	1 (25.0)	14 (50.0)	60 (64.5)
Current	25 (20.0)	2 (50.0)	6 (21.4)	17 (18.3)
Past	25 (20.0)	1 (25.0)	8 (28.6)	16 (17.2)
IV tPA, *n* (%)	72 (46.5)	2 (40.0)	13 (38.2)	57 (49.1)
Taking anti-platelets at admission, *n* (%)	54 (34.8)	4 (80.0)	11 (32.4)	39 (33.6)
Taking anti-coagulants at admission, *n* (%)	35 (22.6)	2 (40.0)	7 (20.6)	26 (22.4)
Pre-stroke mRS ≥ 3, *n* (%)	11 (7.5)	2 (40.0)	3 (9.1)	6 (5.6)
Mean NIHSS (SD)	10.4 (6.4)	13.0 (5.6)	10.0 (5.1)	10.4 (6.8)
Baseline ASPECT score, median [25th, 75th percentile]	8.5 [7.0, 9.0]	6.0 [6.0. 9.0]	8.0 [7.0, 9.0]	9.0 [8.0, 9.0]
General anesthesia, *n* (%)	71 (45.8)	1 (20.0)	13 (38.2)	57 (49.1)
*Occlusion location, n (%)*^*1*^				
M2	145 (93.5)	5 (100.0)	32 (94.1)	108 (93.1)
M3	8 (5.2)	0 (0.0)	2 (5.9)	6 (5.2)
A1	1 (0.6)	0 (0.0)	0 (0.0)	1 (0.9)
A2	1 (0.6)	0 (0.0)	0 (0.0)	1 (0.9)
**Time metrics**				
Wake-up stroke, *n* (%)	29 (19.2)	2 (40.0)	7 (20.6)	20 (17.9)
Time last known well to treating hospital admission (hours), median [p25, p75]^2^	3.8 [1.6,7.2]	3.9 [3.7,5.2]	4.2 [2.6,10.4]	3.4 [1.4,7.1]
Time last known well to imaging (hours), median [p25, p75]	3.1 [1.3,6.8]	4.0 [3.8,5.6]	4.5 [2.0,9.9]	2.7 [1.1,6.6]
Time last known well to groin puncture (hours), median [p25, p75]	4.5 [3.0,8.6]	5.1 [4.3,6.9]	5.8 [3.8,11.4]	4.3 [2.7,8.3]
Time from groin puncture to end of procedure (minutes), median [p25, p75]	31.0 [21.0,45.0]	32.0 [28.0,36.0]	28.5 [17.0,45.0]	31.0 [22.0,48.0]

^1^ Core lab reported, except for where core lab reported CICA or was missing. In those cases, site-reported was used, ^2^ If admitted to 2nd hospital, time is calculated from time of admission to 2nd hospital. Otherwise admission to 1st hospital date/time is used. If subject already in hospital when they started showing symptoms, time was set to missing

Mean NIHSS at presentation was 10.4 (SD 6.4) and 92.5% of the patients were functionally independent (mRS ≤ 2) prior to the stroke. Patients were admitted to the treating hospital on median 3.8 h after last seen well (LSW) (25th, 75th percentile 1.6, 7.2) and received baseline imaging 3.1 (25th, 75th percentile 1.3, 6.8) hours after LSW. Occlusion location was the M2 in 145 case (93.5%), the M3 in 8 cases (5.2%) and the A1 and A2 in 1 case respectively (0.6%). Median baseline Alberta Stroke Program early CT score (ASPECTS) was 8.5 (25th, 75th percentile 7, 9).

Of the 155 patients included in our analysis, 116 (74.8%) were treated with CA, 34 (21.9%) with DA and 5 (3.2%) with a SR plus BGC. Forty-three 43 (27.7%) subjects had both a Trevo (XP or NXT) 3 mm device and a Catalyst 5 used on the 1st pass. Due to the low number of patients in the SR plus BGC arm, all further analysis was only carried out within the CA and DA arm. Intravenous lysis was used in 13 cases of the DA group (38.2%) and in 57 (49.1%) of the CA group.

Procedural and patient outcomes are summarized in Table 2. The primary endpoint (first pass eTICI of 2c or 3) was achieved in 66 (43.1%) of the patients in the overall cohort. Rate of first pass eTICI 2c or 3 was not significantly different between DA (36.4%) and CA (45.2%; *p* = 0.30).

**Table 2:** Procedural and patient outcomes

	Overall (*n* = 155)	SR + BGC (*n* = 5)	Direct aspiration (DA) (*n* = 34)	Combined approach (CA) (*n* = 116)	*p*-value comparing DA and CA
First pass eTICI ≥ 2b50, *n* (%)	107 (69.9)	3 (60.0)	25 (75.8)	79 (68.7)	0.42
First pass eTICI ≥ 2c, *n* (%)	66 (43.1)	2 (40.0)	12 (36.4)	52 (45.2)	0.30
First pass eTICI = 3, *n* (%)	35 (22.9)	2 (40.0)	7 (21.2)	26 (22.6)	0.89
Final pass eTICI ≥ 2b50, *n* (%)	144 (93.5)	5 (100.0)	32 (94.1)	107 (93.0)	0.76
Final pass eTICI ≥ 2c, *n* (%)	92 (59.7)	3 (60.0)	19 (55.9)	70 (60.9)	0.67
Final pass eTICI = 3, *n* (%)	46 (29.9)	2 (40.0)	11 (32.4)	33 (28.7)	0.80
*Number of passes for treatment of target occlusion, n (%)*	*–*				*0.06*
1	93 (60.0)	4 (80.0)	18 (52.9)	71 (61.2)	–
2	40 (25.8)	0 (0.0)	7 (20.6)	33 (28.5)	
≥ 3	22 (14.2)	1 (20.0)	9 (26.5)	12 (10.3)	
Rescue, *n* (%)^1^	24 (15.5)	0 (0.0)	11 (32.4)	13 (11.2)	0.06
Device malfunction, *n* (%)	1 (0.6)	0 (0.0)	0 (0.0)	1 (0.9)	0.99^1^
NIHSS at 24 h, mean (SD)	5.9 (6.4)	6.8 (3.3)	5.8 (6.7)	5.9 (6.4)	0.84
*90 days mRS, n (%)*					
0–2	93 (65.0)	3 (60.0)	20 (64.5)	70 (65.4)	0.93
0–1	77 (53.9)	3 (60.0)	18 (58.1)	56 (52.3)	0.57

^1^ *P*-value adjusted for site could not be calculated due to zero cell count. Unadjusted *p*-value using Fisher’s Exact Test are presented. Interpret with caution, as unadjusted *p*-values are smaller (more significant) than adjusted *p*-values

Final eTICI score of 2c or 3 (55.9% in the DA group vs 60.9% in the SRC group; *p* = 0.67) and first pass eTICI score of 2b or better (75.8% in the DA group vs 68.7% in the CA group; *p* = 0.42) was also not significantly different between groups. The proportion of patients achieving favorable outcome (mRS 0–2) was 65.0% in the overall cohort and 64.5% in the DA group and 65.4% in the CA group. The mean NIHSS at 24 h did not differ significantly between the groups, with mean 5.9 (SD 6.4) in the overall cohort and 5.8 (6.7) in the DA group and 5.9 (6.4) in the CA group.

Safety outcomes are summarized in Table 3. The rate of all-cause mortality at 90 days was 9% and sICH up to 48 h post procedure was 1.3% and did not differ significantly between groups. Device and/or procedure related SAEs at up to 90 days were also not significantly different between both groups (5.9% in the DA group vs 6.0% in the CA group; *p* = 0.98). One perforation was observed in the CA Group.

**Table 3:** Safety outcomes

	Overall (*n* = 155)	SR plus BGC (*n* = 5)	Direct Aspiration (DA) (*n* = 34)	Combined approach (CA) (*n* = 116)	*p*-value comparing DA and CA
All-cause Mortality at 90 days (± 14 days), *n* (%)	14 (9.0)	0 (0.0)	4 (11.8)	10 (8.6)	0.55
In-hospital mortality, *n* (%)	3 (1.9)	0 (0.0)	1 (2.9)	2 (1.7)	0.67
Device and/or procedure related SAEs at 90 days (± 14 days), *n* (%)	9 (5.8)	0 (0.0)	2 (5.9)	7 (6.0)	0.98
Any intracranial hemorrhage, *n* (%)	45 (30.0)	1 (20.0)	7 (20.6)	37 (33.3)	0.01
Symptomatic ICH up to 48 h post-procedure, *n* (%)	2 (1.3)	0 (0.0)	1 (2.9)	1 (0.9)	0.38

## Discussion

In this paper we present the results of the ASSIST registry, in patients treated for a distal occlusion with the Trevo 3 mm device and/or with the Catalyst 5 on the first pass. In contrast to the analysis of the full ASSIST data set we were not able to find a modifying effect of the chosen frontline technique on reperfusion results [11]. However, our analysis was not powered to detect such an effect and therefore the results must be interpreted with caution. It is noteworthy that the observed rate of first pass eTICI 2 c or 3 (43.1%) in this cohort did not appear to differ from the rate observed in the full dataset (42.4%). They are also in line with recently reported results from a randomized controlled trial evaluating the pRESET (43.7%) and Solitaire device (44.3%) in large vessel occlusions, which is encouraging [12]. The safety data with a low rate of sICH (1.3%) and device/procedure-related SAEs (6%) is encouraging and suggest that the Trevo 3 mm and/or the Catalyst 5 aspiration catheter are suitable devices for MDVOs in a sample comprised of patients with distal occlusions, primarily in the M2 location.

To our knowledge the only other data set containing core lab adjudicated reperfusion results for M2 occlusions stems from the HERMES collaboration. They only included the final modified thrombolysis in cerebral infarction (mTICI) 2b or better score and reported a rate of 59.2%, which was lower than the 96.2% reported in the initial ASSIST data set. While these results suggest potential improvements in techniques and materials, compared to the 2015 randomized-controlled trials it must be interpreted with caution as in the ASSIST registry enrollment could have happened up to 48 h post procedure (i.e., patients with poor results may not have been enrolled, thus with the possibility of selection bias). A recent meta-analysis using unadjudicated retrospective papers has reported a rate of first pass eTICI 2c or 3 of 42.5% in 220 patients with primarily distal occlusions, which is comparable to the 43.1% observed in our cohort [13]. Comparison with other retrospective cohorts is complicated as the reperfusion results were physician-self reported and not core lab evaluated and thus could be associated with reperfusion rate overestimation.

Reperfusion success at the first pass (defined as an eTICI 2c or above) is correlated with improved clinical outcomes and reduced complications [14]. While the observed rate of 43.1% is encouraging, there is clearly room for advancements which could be driven either by improved techniques for the treatment of MDVOs, but also by superior materials dedicated to the treatment of MDVOs.

Possible improvements could stem from using smaller and softer materials (i.e., aspiration catheter and microcatheter), which could be associated with less straightening of the vessels. This is important from a safety standpoint as perforations are more likely to happen during EVT of MDVOs than LVOs and this is a possible mechanism for a vessel perforation [15]. Technical aspects which could improve reperfusion are the use of the QUATTRO technique which uses a quadriaxial approach and allows for the entrapment of the clot prior to its extraction on “large bore aspiration catheter” similar to the SAVE technique [16]. Possible advantages over the blind mini-pinning technique [17], those derive from the creation of multiple “joints” in the quadriaxial thrombectomy system and are: the reduction of the need for oversizing the aspiration catheter, the prevention of vessel straightening, avulsion of small perforators and limiting the collapse of smaller arteries. In theory this should be associated with less complications, however this has to be shown in prospective trials addressing this issue specifically in MDVOs.

The study has diverse limitations which are intrinsic to a subgroup-analysis and are associated with the limitations of the main ASSIST paper. Briefly, the most important limitations are (a) patients were not randomized to a strategy but it was chosen based on the preference of the treating center, (b) some patients and their legally authorized representatives may have been consented retrospectively after completion of the procedure, leading possibly to a selection bias, and (c) the main study was not powered for this analysis and therefore the absence of an effect could be due to insufficient statistical power.

## Conclusion

The data suggests that the Trevo 3 mm SR and/or the AXS Catalyst 5 may be an option to treat medium distal vessel occlusion, but more data is needed to demonstrate safety and efficacy in this patient cohort. Further improvements are needed regarding materials and techniques to improve reperfusion results in this patient cohort in the future.

## References

[1] Jovin TG, Li C, Wu L, Wu C, Chen J, Jiang C, Shi Z, Gao Z, Song C, Chen W, et al. Trial of Thrombectomy 6 to 24 Hours after Stroke Due to Basilar-Artery Occlusion. n Engl J Med. 2022;387:1373–84. 10.1056/NEJMoa2207576. 36239645 10.1056/NEJMoa2207576

[2] Goyal M, Menon BK, Van Zwam WH, Dippel DWJ, Mitchell PJ, Demchuk AM, Dávalos A, Majoie CBLM, Van Der Lugt A, De Miquel MA, et al. Endovascular thrombectomy after large-vessel ischaemic stroke: A meta-analysis of individual patient data from five randomised trials. Lancet. 2016;387:1723–31. 10.1016/S0140-6736(16)00163-X. 26898852 10.1016/S0140-6736(16)00163-X

[3] Menon BK, Hill MD, Davalos A, Roos YBWEM, Campbell BCV, Dippel DWJ, Guillemin F, Saver JL, Van Der Lugt A, Demchuk AM, et al. Efficacy of endovascular thrombectomy in patients with M2 segment middle cerebral artery occlusions: Meta-analysis of data from the HERMES Collaboration. J NeuroIntervent Surg. 2019;11:1065–9. 10.1136/neurintsurg-2018-014678. 10.1136/neurintsurg-2018-014678 30975736

[4] Turc G, Bhogal P, Fischer U, Khatri P, Lobotesis K, Mazighi M, Schellinger PD, Toni D, de Vries J, White P, et al. European Stroke Organisation (ESO)—European Society for Minimally Invasive Neurological Therapy (ESMINT) Guidelines on Mechanical Thrombectomy in Acute Ischaemic StrokeEndorsed by Stroke Alliance for Europe (SAFE). Eur Stroke J. 2019;4:6–12. 10.1177/2396987319832140. 31165090 10.1177/2396987319832140 PMC6533858

[5] Powers WJ, Rabinstein AA, Ackerson T, Adeoye OM, Bambakidis NC, Becker K, Biller J, Brown M, Demaerschalk BM, Hoh B, et al. Guidelines for the early management of patients with acute ischemic stroke: 2019 update to the 2018 guidelines for the early management of acute ischemic stroke a guideline for healthcare professionals from the American Heart Association/American Stroke Association. Stroke. 2019;50:E344–E418. 10.1161/STR.0000000000000211. 31662037 10.1161/STR.0000000000000211

[6] Meyer L, Stracke P, Broocks G, Elsharkawy M, Sporns P, Piechowiak EI, Kaesmacher J, Maegerlein C, Hernandez Petzsche MR, Zimmermann H, et al. Thrombectomy versus Medical Management for Isolated Anterior Cerebral Artery Stroke: An International Multicenter Registry Study. Radiology. 2023;307:e220229. 10.1148/radiol.220229. 36786705 10.1148/radiol.220229

[7] Nguyen TN, Qureshi MM, Strambo D, Strbian D, Räty S, Herweh C, Abdalkader M, Olive-Gadea M, Ribo M, Psychogios M, et al. Endovascular Versus Medical Management of Posterior Cerebral Artery Occlusion Stroke: The PLATO Study. Stroke. 2023; 10.1161/strokeaha.123.042674. 37222709 10.1161/STROKEAHA.123.042674

[8] Meyer L, Stracke CP, Jungi N, Wallocha M, Broocks G, Sporns PB, Maegerlein C, Dorn F, Zimmermann H, Naziri W, et al. Thrombectomy for Primary Distal Posterior Cerebral Artery Occlusion Stroke: The TOPMOST Study. JAMA Neurol. 2021;78:434–44. 10.1001/jamaneurol.2021.0001. 33616642 10.1001/jamaneurol.2021.0001 PMC7900924

[9] Saber H, Desai SM, Haussen D, Al-bayati A, Majidi S, Mocco J, Hassan AE, Rajah G, Waqas M, Davies JM, et al. Endovascular Therapy vs Medical Management for Patients With Acute Stroke With Medium Vessel Occlusion in the Anterior Circulation. JAMA Netw Open. 2022;5:e2238154–e2238154. 10.1001/jamanetworkopen.2022.38154. 36279137 10.1001/jamanetworkopen.2022.38154 PMC9593229

[10] Saver JL, Chapot R, Agid R, Hassan A, Jadhav AP, Liebeskind DS, Lobotesis K, Meila D, Meyer L, Raphaeli G, et al. Thrombectomy for Distal, Medium Vessel Occlusions: A Consensus Statement on Present Knowledge and Promising Directions. Stroke. 2020;51:2872–84. 10.1161/strokeaha.120.028956. 32757757 10.1161/STROKEAHA.120.028956

[11] Gupta R, Miralbés S, Calleja Bonilla A, Naravetla B, Majjhoo AQ, Rayes M, Spiotta AM, Loehr C, Cioltan A, Vollherbst DF, et al. Technique and impact on first pass effect primary results of the ASSIST global registry. J Neurointerv Surg. 2024; 10.1136/jnis-2023-021126. 38195248 10.1136/jnis-2023-021126 PMC11877071

[12] Nogueira RG, Lobsien D, Klisch J, Pielenz D, Lobsien E, Sauvageau E, Aghaebrahim N, Möhlenbruch M, Vollherbst D, Ulfert C, et al. Thrombectomy With the pRESET vs Solitaire Stent Retrievers as First-Line Large Vessel Occlusion Stroke Treatment: A Randomized Clinical Trial. JAMA Neurol. 2024;81:170–8. 10.1001/jamaneurol.2023.5010. 38165690 10.1001/jamaneurol.2023.5010 PMC10762632

[13] Toh KZX, Koh MY, Loh EW, Kwok GYR, Teo YH, Teo YN, Goh CXY, Syn NLX, Ho AFW, Sia CH, et al. Distal medium vessel occlusions in acute ischaemic stroke—Stent retriever versus direct aspiration: A systematic review and meta-analysis. Eur Stroke J. 2023;8:434–47. 10.1177/23969873231151262. 37231692 10.1177/23969873231151262 PMC10334182

[14] Gaurav J, De Paula HC, Aaron W, Elizabeth L, Varun N, Ryan TM, Marcella W, Ravi S, Carolyn AC, Chad S, et al. Beyond the first pass: revascularization remains critical in stroke thrombectomy. J NeuroIntervent Surg. 2019;11:1095. 10.1136/neurintsurg-2019-014773. 10.1136/neurintsurg-2019-014773 31048458

[15] Schulze-Zachau V, Brehm A, Ntoulias N, Krug N, Tsogkas I, Blackham KA, Möhlenbruch MA, Jesser J, Cervo A, Kreiser K, et al. Incidence and outcome of perforations during medium vessel occlusion compared with large vessel occlusion thrombectomy. J Neurointerv Surg. 2023; 10.1136/jnis-2023-020531. 10.1136/jnis-2023-020531 37524518

[16] Psychogios MN, Tsogkas I, Blackham K, Schulze-Zachau V, Rusche T, Ntoulias N, Brehm A, Fischer U, Sporns PB. The Quattro Technique for Medium Distal Vessel Occlusion Stroke. Clin Neuroradiol. 2023; 10.1007/s00062-023-01317-8. 37378841 10.1007/s00062-023-01317-8

[17] Pérez-García C, Moreu M, Rosati S, Simal P, Egido JA, Gomez-Escalonilla C, Arrazola J. Mechanical Thrombectomy in Medium Vessel Occlusions: Blind Exchange With Mini-Pinning Technique Versus Mini Stent Retriever Alone. Stroke. 2020;51:3224–31. 10.1161/strokeaha.120.030815. 33070712 10.1161/STROKEAHA.120.030815

